# Food Insecurity Is Associated With Current Asthma, Wheeze, and Lung Function in Children and Adults

**DOI:** 10.1111/crj.70052

**Published:** 2025-02-10

**Authors:** Weiliang Kong, Yilian Xie, Kunlong Xiong, Jingjing Hu, Weina Huang, Chao Cao

**Affiliations:** ^1^ Department of Respiratory and Critical Care Medicine, key Laboratory of Respiratory Disease of Ningbo First Affiliated Hospital of Ningbo University Ningbo China; ^2^ Department of Hepatology First Affiliated Hospital of Ningbo University Ningbo China

**Keywords:** asthma, FeNO, food insecurity, lung function, NHANES, wheeze

## Abstract

**Background:**

Food insecurity (FI) has been a global threat, as a social determinant in connection with the prevalence of diseases requiring dietary interventions, such as asthma, has been established. This study aims to examine the relationship between FI and respiratory health outcomes.

**Methods:**

This cross‐sectional study included 7626 children and 17 530 adults from the 2007 to 2012 National Health and Nutritional Examination Surveys (NHANES) in the United States. Weighted multivariate regression models were used to evaluate the associations between FI and respiratory outcomes, including current asthma, wheezing, fractional exhaled nitric oxide (FeNO), and lung function.

**Results:**

The weighted prevalence of high FI was 19.68% in children and 13.74% in adults. In adults, high FI was significantly associated with current asthma (OR: 1.41, 95% CI: 1.19–1.67) and wheezing (OR: 1.72, 95% CI: 1.48–1.99). The association with asthma was stronger in women (*p* for interaction = 0.02) and non‐Hispanic Whites (*p* for interaction = 0.04), whereas wheezing showed stronger associations in non‐Hispanic Whites (*p* for interaction = 0.01). High FI was linked to lower percent‐predicted forced expiratory volume in 1 s (FEV_1_) in children (β: −15.93%, 95% CI: −27.82%, −4.03%) and adults (β: −1.13%, 95% CI: −2.22%, −0.04%) without asthma or wheezing. Additionally, high FI was inversely associated with FeNO in adults with current asthma (β: −3.36, 95% CI: −5.54, −1.17) and wheezing (β: −4.40, 95% CI: −7.79, −1.02).

**Conclusions:**

FI is associated with increased asthma and wheezing in adults, particularly among women and non‐Hispanic Whites, and with reduced FEV1 in both adults and children without asthma and wheezing.

## Graphical abstract

FI is associated with asthma and wheeze in adults and with FEV1 in children and adults without asthma and wheeze. FI does not seem to cause airway allergic reactions, because households with high FI had lower FeNO measurements in adults with current asthma or wheeze.

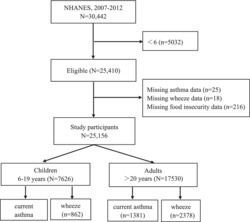

**Abbreviations**
BMI: body mass index (weight [kg]/height[m]^2^)
FeNO: fractional exhaled nitric oxide
FEV_1_
: forced expiratory volume in 1 s
FI: food insecurity
FVC: forced vital capacity
NHANES: National Health and Nutrition Examination Survey

## 1 Introduction

Asthma is a prevalent allergic inflammatory disease characterized by symptoms such as coughing, wheezing, shortness of breath, and chest tightness [1]. The etiology and pathogenesis of asthma are multifactorial, driven by both genetic and environmental factors, with environmental exposures playing a particularly significant role. Key environmental triggers include allergens, infections, tobacco smoke, air pollution, and dietary factors [2]. Among these, diet is a modifiable risk factor [3], and aside from food allergens, research has shown that certain nutrients can influence immune responses related to asthma [4, 5, 6, 7, 8]. Consequently, diet has garnered increasing attention for its potential role in asthma development and management.

Food insecurity (FI), defined as inadequate access to safe and nutritious food, presents an additional challenge for individuals with asthma and is a major public health concern in many countries, including the United States [9]. This is especially true for those managing food allergies [10]. FI often leads to a reliance on energy‐dense, nutrient‐poor foods, which may exacerbate respiratory conditions. Households facing FI also experience increased financial strain, particularly when managing food allergies, which require the avoidance of specific allergens. This economic burden disproportionately affects low‐income families, making it difficult to afford safe, allergen‐free foods [11, 12, 13]. Despite the potential impact of FI on respiratory health, screening for FI in asthma patients remains limited. A 2020 survey by the American Academy of Allergy, Asthma, and Immunology found that only 24.5% of allergy providers had screened for FI, and 71.2% were unaware of whether their patients experienced FI [14].

The association between FI, nutrient deficiencies, food allergies, and asthma has been explored in various studies, but results have been inconsistent due to differing definitions of FI and limited study populations [15, 16, 17]. Despite the importance of this issue, there has been a lack of attention to FI in asthma management and few relevant studies in the general US population [14]. Given the significant policy and clinical implications, a comprehensive understanding of the relationship between FI and respiratory diseases is essential. Therefore, we conducted an analysis of data from the National Health and Nutrition Examination Survey (NHANES) in the United States to evaluate the potential association between FI and several respiratory outcomes, including asthma, wheezing, fractional exhaled nitric oxide (FeNO), and lung function.

## 2 Methods

### 2.1 Design

**FIGURE 1:**
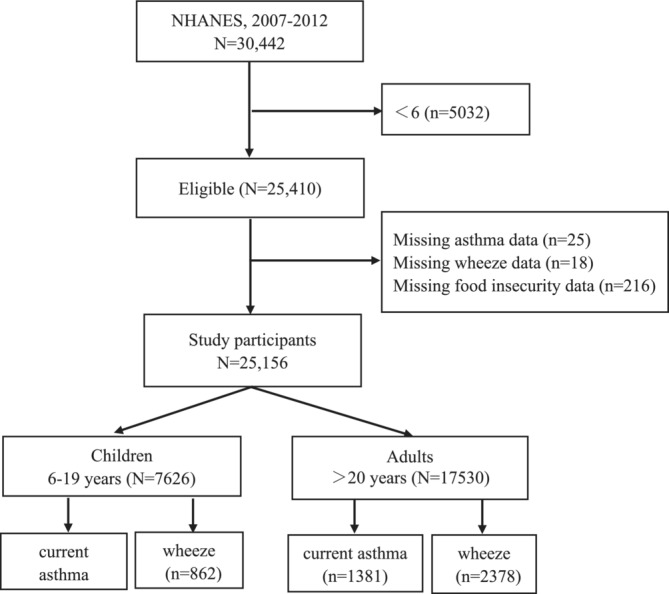
Participants' flowchart.

This retrospective, cross‐sectional study analyzed data from the NHANES 2007–2012 cycles, which included children (aged 6–19 years) and adults (aged > 20 years) who responded to questions on demographic, socioeconomic, dietary, and health‐related factors. After applying the inclusion and exclusion criteria, a sample of 7626 children and 17 530 adults remained. The study flowchart is shown in Figure 1.

The NHANES employs a stratified, multistage, probability‐cluster sampling method designed to collect a representative sample of data on health and nutrition in the United States. These data are publicly available at CDC.gov. The detailed methods and protocols of the NHANES study were approved by the CDC/NCHS Research Ethics Review Board, and informed consent was obtained from all participants. This study was exempt from human‐subject ethical review because all the data we used were freely available to the public.

### 2.2 FI

Food security refers to having access at all times to enough food for an active, healthy life. In contrast, FI occurs when this access is limited or uncertain [18]. In this study, FI was assessed using the US Department of Agriculture (USDA) Food Security Survey Module (FSSM) of the NHANES.

The FSSM questionnaire consists of 18 items for households with children under the age of 18, but households without children are presented with 10 items. These items primarily pertain to the household's capacity to acquire adequate, safe, and nutritious food during the preceding year, as well as the level of confidence that all members display in their ability to do so. Based on the three‐stage screening survey, household food security was categorized into four levels: full (no affirmative response in any of the items), marginal (1–2 affirmative responses), low (3–5 affirmative responses for households without children and 3–7 affirmative responses for households with children under the age of 18 years), and very low (6–10 affirmative responses for households without children and 8–18 affirmative responses for households with children under the age of 18 years). For the purposes of this study, households with low or very low food security were classified as having high FI, whereas those with high or marginal food security were categorized as having low FI [18].

### 2.3 Current Asthma and Wheezing

We examined two outcomes related to respiratory health: current asthma and wheezing. Current asthma was defined based on self‐reported responses to the following questions: “Has a doctor or other health professional ever told you that you have asthma?” and “Do you still have asthma?” Participants who answered “yes” to both questions were classified as having current asthma. Wheezing was defined as a positive response to the question “In the past 12 months, have you had wheezing or whistling in your chest?” Participants without these conditions were included as controls, following the exclusion of those with missing data.

### 2.4 Lung Function and FeNO

Survey participants who were eligible for this study and the spirometry component were aged 6–79 years and excluded those who had current chest pain and physical problems with forceful expiration, were taking supplemental oxygen, had recent surgery of the eye, recent surgery of the chest or abdomen, recent heart attack, stroke, or tuberculosis exposure, had recently coughed up blood, or had any painful ear infection. Spirometry testing followed the method recommended by the American Thoracic Society (ATS) [19]. Participants whose pre‐bronchodilator forced expiratory volume in 1 s/forced vital capacity (FEV_1_/FVC) ratio was below the lower limit of normal and/or below 70% of the predicted value for their demographic characteristics underwent a repeat bronchial dilation test. An A–F [19] grade was used to rate the quality of the measurements of FEV_1_ and FVC. Only the reliable grades (A and B) and the best FEV_1_, FVC, and FEV_1_/FVC spirometry results were included in this analysis.

Several prediction equations based on age, sex, height, and race have been developed to predict lung function values. To maintain the consistency of the measurement methods, we chose the Hankinson equation [20], which was derived from NHANES III data. We generated predicted values of FEV_1_, FVC, and FEV_1_/FVC [20], and the spirometry data were converted into percentages of the predicted values.

Following the NHANES protocol, the participants were coached by the health technicians to maintain their airflow at a suitable rate during testing of the FeNO. Measurements were obtained using the Aerocrine NIOX MINO device. Two valid and reproducible FeNO measurements were required, and the result was reported as the mean. Valid and reproducible measurements were defined as follows: Either or both of the two FeNO measurements was < 30 ppb and the difference was < 2 ppb, both measurements were > 30 ppb and the difference was < 10%, or both values were below the limit of detection. If the first two exhalations were not reproducible, no more than two additional exhalations were performed.

### 2.5 Covariates

Potential confounding variables were selected based on previous associations found in the literature and adjusted for in the multivariate models. These covariates were sociodemographic characteristics such as sex, age, race (non‐Hispanic White, non‐Hispanic Black, Mexican American, and others), household income (≥ $20 000/year, < $20 000/year), insured status, body mass index (BMI), family history of asthma, tobacco exposure (defined by serum cotinine), smoker status (defined by self‐reported questionnaires in adults), and medication (oral glucocorticoids and inhaled bronchodilators).

Using the CDC growth charts for ages 2–20 years [21], the BMI in children was categorized as indicating underweight (< 5th percentile), normal weight (5th–85th percentile), overweight (85th–95th percentile), and obesity (≥ 95th percentile). The adult BMI data were classified according to the World Health Organization (WHO) system as follows: underweight, < 18.5 kg/m^2^; normal range, 18.5–24.9 kg/m^2^; overweight, 25–30 kg/m^2^; and obesity, > 30 kg/m^2^.

The assessment of tobacco exposure was based on serum cotinine concentration, and participants were classified into three categories: no exposure (< 0.015 ng/mL), second‐hand smoke exposure (≥ 0.015 ng/mL but < 10 ng/mL), and active smoker (≥ 10 ng/mL) [22, 23]. Smoker status was assessed by self‐reported questionnaires only in adults, who were asked the following questions: “Have you smoked at least 100 cigarettes in your life?” and “Do you smoke cigarettes now?” Status was classified as follows: active smoker (two positive answers), former smoker (a positive answer to the former question only), and nonsmoker (two negative answers).

### 2.6 Statistical Analysis

The NHANES is a population‐based survey that uses a nonrandom sampling design to ensure the representation of specific population subgroups and assigns sample weights to respondents to account for nonresponse and stratification, among other complexities of the survey design [24]. Following the official guidance of the NHANES, we merged data from the three NHANES cycles (2007–2012) into one 6‐year pooled dataset. A Taylor‐series linearization was used to approximate the standard errors (SEs) of all continuous variables. Student's *t*‐test was used to study their associations. Weighted %, means with 95% CI, and survey‐weighted chi‐square tests were used to study the associations of the categorical variables. We used multiple logistic regression models to assess the associations between FI and current asthma among children and adults. The same methods were used to assess the associations among subgroups of participants with current asthma in stratified and interaction analyses. We also used multiple linear regression models to assess the relationships among FI, abnormal spirometry results, and FeNO. Data analysis was performed using R (Version 4.2.0, “survey” package).

## 3 Results

The analysis included 25 156 participants, comprising 7626 children and 17 530 adults. The weighted prevalence of high FI was 19.68% in children and 13.74% in adults. Compared with low FI, children and adults with high FI were more likely to be non‐Hispanic Black and Mexican Americans, have lower annual household income, have lower best FEV_1_ and FVC, have more family history of asthma, have more wheezing, have more current asthma, have less insurance, and were unlikely to be active smokers or have active tobacco exposure. In addition, the prevalence of high FI was greater in older adults. Additional demographic and behavioral characteristics are presented in Table 1.

**TABLE 1:** Characteristic of the study participants by FI in children and adults.

Variables	Children				Adults			
	Total	Low FI	High FI	*p*	Total	Low FI	High FI	*p*
	(*n* = 7626)	(*n* = 5593)	(*n* = 2033)		(*n* = 17 530)	(*n* = 14 251)	(*n* = 3279)	
Food insecurity	19.68 (0.01)	NA	NA		13.74 (0.01)	NA	NA	
Age, year	12.53 (0.07)	12.54 (0.08)	12.43 (0.11)	0.39	47.04 (0.36)	48.00 (0.37)	41.28 (0.52)	< 0.001
Age				0.95				< 0.001
5–11	41.92 (0.02)	41.93 (0.95)	42.06 (1.51)			NA	NA	
12–19	58.08 (0.02)	58.07 (0.95)	57.94 (1.51)			NA	NA	
20–39	NA	NA	NA		24.72 (0.01)	26.78 (0.68)	12.18 (0.87)	
40–59	NA	NA	NA		38.34 (0.02)	38.33 (0.62)	38.49 (1.51)	
≥ 60	NA	NA	NA		36.93 (0.01)	34.89 (1.00)	49.33 (1.87)	
Sex				0.39				0.37
Female	48.96 (0.02)	49.10 (0.90)	47.57 (1.53)		51.90 (0.02)	51.78 (0.35)	52.82 (1.15)	
Male	51.04 (0.02)	50.90 (0.90)	52.43 (1.53)		48.10 (0.02)	48.22 (0.35)	47.18 (1.15)	
Race and ethnicity				< 0.001				< 0.001
Non‐Hispanic White	57.09 (0.04)	61.41 (2.17)	38.98 (3.39)		67.89 (0.04)	71.48 (1.91)	46.00 (3.51)	
Non‐Hispanic Black	14.65 (0.01)	13.11 (1.34)	21.17 (1.77)		11.40 (0.01)	10.05 (0.90)	19.82 (1.98)	
Mexican American	13.89 (0.01)	11.47 (1.35)	23.98 (2.72)		8.21 (0.01)	6.72 (0.89)	17.26 (2.25)	
Others	14.37 (0.01)	14.01 (1.09)	15.87 (1.99)		12.50 (0.01)	11.75 (0.93)	16.92 (1.80)	
Annual household income				< 0.001				< 0.001
< $20 000/year	15.81 (0.01)	11.47 (0.85)	33.98 (1.52)		15.24 (0.01)	11.75(0.62)	37.54 (1.64)	
≥ $20 000/year	80.93 (0.04)	86.07 (0.98)	63.39 (1.61)		81.34 (0.03)	85.43 (0.66)	59.05 (1.64)	
BMI, m/kg^2^	21.53 (0.11)	21.38 (0.13)	22.09 (0.16)	< 0.001	28.67 (0.10)	28.51 (0.11)	29.64 (0.19)	< 0.001
BMI category				0.004				< 0.001
Underweight	33.39 (0.02)	34.44 (0.87)	29.83 (1.11)		1.60 (0.00)	1.49 (0.14)	2.26 (0.35)	
Normal	33.39 (0.02)	43.73 (0.69)	44.12 (1.11)		29.34 (0.01)	29.89 (0.79)	25.74 (1.12)	
Overweight	13.01 (0.01)	12.88 (0.71)	13.71 (0.94)		33.50 (0.01)	34.07 (0.69)	30.21 (1.03)	
Obesity	8.68 (0.01)	8.11 (0.65)	10.77 (0.96)		34.42 (0.01)	33.43 (0.73)	40.54 (1.14)	
Tobacco exposure				< 0.001				< 0.001
Active smoker	44.55 (0.02)	47.23 (1.22)	33.59 (1.98)		49.45 (0.02)	52.90 (0.97)	28.45 (1.41)	
SHS	31.87 (0.02)	29.50 (1.50)	41.52 (1.92)		20.21 (0.01)	19.91 (0.68)	22.03 (0.99)	
Nonsmoker	7.82 (0.01)	6.96 (0.52)	11.45 (1.20)		24.47 (0.01)	21.52 (0.74)	42.57 (1.65)	
Smoke status								< 0.001
Active smoker	NA	NA	NA		20.94 (0.01)	18.01 (0.63)	39.34 (1.66)	
Former smoker	NA	NA	NA		24.16 (0.01)	25.40 (0.67)	16.47 (0.99)	
Nonsmoker	NA	NA	NA		54.85 (0.02)	56.56 (0.93)	44.08 (1.80)	
Family history of asthma	30.11 (0.02)	29.14 (1.09)	36.79 (1.82)	< 0.001	18.86 (0.01)	18.13 (0.45)	26.01 (1.23)	< 0.001
Insurance	88.57 (0.04)	89.73 (0.97)	83.69 (1.45)	< 0.001	79.91 (0.03)	83.27 (0.61)	59.44 (1.24)	< 0.001
Medication	3.20 (0.00)	3.16 (0.28)	3.50 (0.51)	0.5	3.90 (0.00)	3.85 (0.27)	4.18 (0.49)	0.54
Wheezing	11.66 (0.01)	11.10 (0.72)	14.13 (1.25)	0.04	13.19 (0.01)	11.68 (0.49)	22.62 (1.15)	< 0.001
Current asthma	11.25 (0.01)	10.47 (0.54)	14.76 (0.92)	< 0.001	7.74 (0.01)	7.11 (0.43)	11.46 (0.87)	< 0.001
FeNO, ppb	16.66 (0.36)	16.62 (0.37)	16.82 (0.64)	0.75	16.64 (0.28)	16.98 (0.31)	14.50 (0.47)	< 0.001
Best FEV_1_, mL	2814.14 (22.00)	2830.69 (25.29)	2739.05 (33.52)	0.03	3226.62 (13.87)	3235.23 (15.63)	3168.21 (27.38)	0.04
Best FVC, mL	3268.40 (26.79)	3288.66 (30.34)	3174.18 (37.81)	0.01	4120.16 (15.38)	4137.91 (17.58)	4005.52 (34.26)	0.002
Best FEV_1_/FVC, %	86.51 (0.12)	86.48 (0.14)	86.68 (0.24)	0.45	78.34 (0.19)	78.19 (0.19)	79.21 (0.38)	0.004

*Note:* Weighted mean +/− SE and Student's *t*‐test for continuous variables. Weighted %, mean (95% CI), and Cochran–Mantel–Haenszel chi‐square test for categorical variables.

Table 2 presents the associations among FI, current asthma, and wheezing in children and adults. After adjusting for all selected covariates by multiple logistic regression (Model 3), adult participants with high FI had a 72% greater risk of wheeze compared with low (OR: 1.72, 95% CI: 1.48–1.99, *p* < 0.001) and a 41% greater risk of current asthma (OR: 1.41, 95% CI: 1.19–1.67, *p* < 0.0001). Children so compared showed a trend to more prevalent wheeze (OR: 1.21, 95% CI: 0.90–1.62, *p* = 0.20) and current asthma (OR: 1.27, 95% CI: 0.99–1.63, *p* = 0.06). Stratified and interaction analyses showed whether the associations of FI versus wheeze/current asthma were modified by selected covariates in adults (Table 3) and children (Table 4). The results showed that the associations with wheezing and asthma were enhanced in non‐Hispanic White adults (*p* = 0.01 and 0.04, respectively). We also noted a strong association between FI and current asthma in women (*p* = 0.02).

**TABLE 2:** Odds ratios (95%CI) of the associations between FI and wheeze/current asthma.

	Children						Adults					
	Model 1	*p*	Model 2	*p*	Model 3	*p*	Model 1	*p*	Model 2	*p*	Model 3	*p*
Asthma	1.48 (1.21, 1.80)	< 0.001	1.50 (1.22, 1.84)	< 0.001	1.27 (0.99, 1.63)	0.06	1.69 (1.39, 2.05)	< 0.001	1.85 (1.54, 2.22)	< 0.001	1.41 (1.19, 1.67)	< 0.001
Wheeze	1.32 (1.02, 1.71)	0.04	1.38 (1.05, 1.80)	0.02	1.21 (0.90, 1.62)	0.20	2.21 (1.95, 2.50)	< 0.001	2.49 (2.19, 2.83)	< 0.001	1.72 (1.48, 1.99)	< 0.001

*Note:* Model 1 adjusted for none. Model 2 adjusted for sex, age, and race. Model 3 additionally adjusted for annual household income, insurance, family history of asthma, BMI, tobacco exposure, smoke status (adults), and medication.

**TABLE 3:** The associations stratified by subgroups in adults.

Variables	Wheezing		Current asthma	
	OR (95% CI)	*p*	OR (95% CI)	*p*
Age		0.18		0.88
20–59	1.76 (1.33, 2.33)		1.63 (1.16, 2.29)	
≥ 60	1.70 (1.42, 2.03)		1.35 (1.10, 1.65)	
Sex		0.11		0.02
Female	1.89 (1.55, 2.30)		1.54 (1.26, 1.89)	
Male	1.48 (1.20, 1.81)		1.20 (0.90, 1.59)	
Race		0.01		0.04
Non‐Hispanic White	1.92 (1.56, 2.36)		1.70 (1.32, 2.19)	
Others	1.42 (1.19, 1.70)		1.10 (0.90, 1.33)	
BMI		0.33		0.91
Underweight and normal	1.38 (1.02, 1.85)		1.51 (1.10, 2.08)	
Overweight and obesity	1.85 (1.57, 2.19)		1.39 (1.13, 1.70)	
Family history of asthma		0.3		1
Yes	1.60 (1.22, 2.10)		1.37 (1.07, 1.75)	
No	1.82 (1.48, 2.23)		1.44 (1.02, 2.02)	
Annual household income		0.66		0.07
≥ $20 000/year	1.89 (1.54, 2.33)		1.63 (1.30, 2.04)	
< $20 000/year	1.69 (1.35, 2.11)		1.23 (0.94, 1.60)	
Insurance		0.1		0.8
Yes	1.88 (1.52, 2.32)		1.40 (1.13, 1.73)	
No	1.40 (1.08, 1.81)		1.46 (1.15, 1.85)	
Active smoker^a^		0.11		0.67
Yes	1.62 (1.31, 2.01)		1.46 (1.08, 1.97)	
No	2.02 (1.67, 2.45)		1.41 (1.10, 1.80)	

Abbreviation: *p* = *p* for interaction.

^a^ Active smoker was defined by cotinine ≥ 10 μg/dL and self‐reported active smoker. All models adjusted for sex, age, race and ethnicity, annual household income, insurance, family history of asthma, BMI, tobacco exposure, smoke status (adults), and medication except for the stratifying variable in each subgroup analysis.

**TABLE 4:** The associations stratified by subgroups in children.

Variables	Wheezing		Current asthma	
	OR (95% CI)	*p*	OR (95% CI)	*p*
Age		0.74		0.87
5–11	1.09 (0.80, 1.49)		1.35 (0.93, 1.95)	
12–19	1.30 (0.90, 1.88)		1.17 (0.90, 1.52)	
Sex		0.54		0.43
Female	1.25 (0.83, 1.87)		1.16 (0.90, 1.50)	
Male	1.16 (0.81, 1.65)		1.37 (0.94, 1.99)	
Race and ethnicity		0.33		0.12
Non‐Hispanic White	1.40 (0.81, 2.41)		1.06 (0.87, 1.29)	
Others	1.05 (0.83, 1.34)		1.62 (1.00, 2.60)	
BMI		0.96		0.17
Underweight and normal	1.19 (0.86, 1.67)		1.40 (1.02, 1.91)	
Overweight and obesity	1.31 (0.77, 2.21)		1.03 (0.73, 1.44)	
Family history of asthma		0.33		0.13
Yes	1.34 (0.87, 2.05)		1.42 (1.02, 1.99)	
No	1.09 (0.71, 1.68)		1.08 (0.79, 1.47)	
Annual household income		0.23		0.07
≥ $20 000/year	0.97 (0.73, 1.29)		1.42 (1.07, 1.88)	
< $20 000/year	1.30 (0.90, 1.86)		0.98 (0.68, 1.42)	
Insurance		0.13		0.07
Yes	1.26 (0.91, 1.74)		1.35 (1.03, 1.76)	
No	0.84 (0.45, 1.57)		0.78 (0.38, 1.57)	
Tobacco exposure		0.5		0.97
Active smoker	1.54 (0.87, 2.73)		1.29 (0.98, 1.69)	
Secondhand smoker and nonsmoker	1.21 (0.88, 1.66)		1.39 (0.77, 2.53)	

*Note:* All models adjusted for sex, age, race and ethnicity, annual household income, insurance, family history of asthma, BMI, tobacco exposure, and medication except for the stratifying variable in each subgroup analysis.

Abbreviation: *p* = *p* for interaction.

Table 5 shows the multiple linear regression results for FI, spirometry, and FeNO. In participants without wheeze or current asthma, high FI was significantly associated with decrements in percent‐of‐predicted FEV_1_ among children (β: −15.85, 95% CI: −26.88 to −4.82) and adults (β: −1.13, 95% CI: −2.22 to −0.04). The observed decrements ranged from 1.13% to 15.85%. In children with current asthma, we observed a significant association between FI and percent‐of‐predicted FVC (β: −2.56, 95% CI: −5.05 to −0.06). In adults with wheeze, we observed a significant association between FI and percent‐of‐predicted FEV_1_/FVC (β: 1.58, 95% CI: 0.28–2.88). Moreover, FI was negatively associated with FeNO in adults with current asthma (β: −4.4, 95% CI: −7.09 to −1.02) or wheeze (β: −3.36, 95% CI: −5.54 to −1.17).

**TABLE 5:** FI and spirometry in children and adults.

Spirometry and FeNO	Children		Adults	
	β (95% CI)	*p*	β (95% CI)	*p*
The general population				
% predicted FEV_1_	−11.14 (−21.20, −1.07)	0.03	−0.6 (−1.70, 0.50)	0.28
% predicted FVC	−0.57 (−1.76, 0.62)	0.34	−0.3 (−1.23, 0.62)	0.51
% predicted FEV_1_/FVC	−0.11 (−0.79, 0.56)	0.74	−0.27 (−1.13, 0.58)	0.52
FeNO, ppb	−0.47 (−2.22, 1.27)	0.58	−1.67 (−2.56, −0.77)	< 0.001
Participants with wheeze				
% predicted FEV_1_	7.49 (−19.21, 34.20)	0.57	1.63 (−1.29, 4.55)	0.26
% predicted FVC	−3.49 (−7.07, 0.09)	0.06	−0.04 (−0.85, 0.77)	0.92
% predicted FEV_1_/FVC	0.72 (−2.05, 3.49)	0.60	1.58 (0.28, 2.88)	0.02
FeNO, ppb	−1.3 (−5.91, 3.30)	0.57	−3.36 (−5.54, −1.17)	0.004
Participants with current asthma				
% predicted FEV_1_	7.32 (−20.45, 35.10)	0.59	1.07 (−2.61, 4.76)	0.56
% predicted FVC	−2.56 (−5.05, −0.06)	0.04	0.58 (−2.55, 3.71)	0.71
% predicted FEV_1_/FVC	−0.71 (−2.96, 1.54)	0.52	0.62 (−2.10, 3.33)	0.65
FeNO, ppb	2.45 (−2.29, 7.20)	0.30	−4.4 (−7.79, −1.02)	0.01
Participants without asthma and wheeze				
% predicted FEV_1_	−15.93 (−27.82, −4.03)	0.01	−1.13 (−2.22, −0.04)	0.04
% predicted FVC	−0.28 (−1.52, 0.97)	0.66	−0.64 (−1.65, 0.36)	0.2
% predicted FEV_1_/FVC	−0.13 (−0.77, 0.51)	0.69	−0.52 (−1.38, 0.34)	0.23
FeNO, ppb	−0.54 (−2.70, 1.62)	0.62	−1.11 (−2.27, 0.04)	0.06

*Note:* All models were adjusted for household income, BMI, family history of asthma, tobacco exposure (children), smoke status (adults), medication, and current asthma (in the general population). FeNO, additionally adjusted for age, sex, race and ethnicity, and medication.

## 4 Discussion

This cross‐sectional study of US children and adults found that high FI was associated with an increased risk of current asthma and wheezing in adults, with these associations being more pronounced in women and non‐Hispanic White individuals. Additionally, among adults with asthma or wheezing, high FI was linked to lower FeNO. In contrast, in individuals without asthma or wheezing, both children and adults with high FI exhibited lower FEV_1_. These findings underscore the respiratory implications of FI and suggest potential areas for public health interventions.

The relationship between FI and health outcomes has been widely explored in numerous studies, particularly its associations with malnutrition [25], obesity [26], and chronic diseases such as diabetes [27] and cardiovascular disease [28]. However, the impact of FI on respiratory conditions, specifically asthma and wheezing, has been less thoroughly investigated. Some studies have demonstrated a significant association between FI and respiratory symptoms or exacerbations in COPD [29]. Adults in high FI households are at greater risk of COPD compared to those in food‐secure households [30]. Additionally, FI has been linked to higher asthma incidence in South Korean adults [31]. A study involving 20 578 kindergarten teachers from the Early Childhood Longitudinal Study‐Kindergarten cohort found a positive association between higher FI and increased asthma (OR: 1.18, 95% CI: 1.17–1.20) [15]. Another study, conducted among 1307 children aged 6–12 years from public schools in Latin America, revealed a significant relationship between FI and moderate asthma (OR: 1.71; 95% CI: 1.01–2.89) as well as severe asthma (OR: 2.51; 95% CI: 1.28–4.93) [17]. Similarly, data from the US kindergarten cohort longitudinal study supported this association in children [32].

Our findings consistently showed an association between high FI and the risk of asthma and wheezing in adults. However, the results were less consistent for children, which may be due to variations in the age ranges of children studied or differences in population selection in previous research. The increased risk of asthma and wheezing in adults with FI could be explained by several potential mechanisms. First, households experiencing FI often have lower socioeconomic status, characterized by reduced household income and increased psychological stress, anxiety, and depression [33]. Chronic stress can induce systemic inflammation [34], exacerbating respiratory problems, including the onset of asthma. Our findings also show that FI is associated with lower levels of FeNO, suggesting that FI may have a potential effect on airway inflammation in individuals with asthma. The exact mechanisms, however, require further exploration. Additionally, individuals in FI households often face barriers to healthcare access, including financial constraints, which may limit their ability to avoid allergens [35] or seek timely medical assistance [36].

Dietary patterns among individuals experiencing FI may further contribute to respiratory health risks. These individuals tend to consume fewer fresh fruits and vegetables while eating more red meat and ultraprocessed foods [14, 37]. Fruits and vegetables are essential sources of key nutrients such as vitamins [38, 39], minerals [40], flavonoids [41], and fibers [42], which play critical roles in maintaining immune defense mechanisms in the respiratory tract. The observed decline in FEV_1_ among both children and adults without asthma and wheezing in FI households may be attributed to the deficiency of these vital nutrients. Moreover, our study identified that the associations between FI and respiratory health outcomes were more pronounced among non‐Hispanic White individuals and females. The heightened vulnerability in females could be linked to sex‐specific factors, including the influence of estrogen and progesterone on airway inflammation and hyperreactivity, which may predispose women to asthma, particularly after puberty. Females may also experience a greater sensitivity to the health effects of FI due to the dual burden of economic stress and societal roles. Although minority groups are generally considered to be at higher risk for adverse respiratory outcomes, our study found a stronger association between FI and asthma among non‐Hispanic White individuals. This may be attributed to differences in social support systems and environmental exposures in this population. Non‐Hispanic Whites might exhibit a more pronounced psychological stress response to economic hardship and FI, which could increase asthma risk and severity through inflammatory pathways.

Our study has two main strengths. First, we used a large, heterogeneous population‐based sample to support evaluation of the relationship between FI and current asthma, wheezing, FeNO, and lung function. Second, a wide range of covariates and potential confounders, including asthma medication, were controlled. The study limitations were as follows: First, this was a cross‐sectional study, and food security may change over time; thus, causal inferences were therefore not possible. Second, some variables were self‐reported, these may have been underdiagnosed or misclassified and lead to biased results. Third, despite the use of the A–E grading system for pulmonary function test completion, a sampling bias may remain, because the excluded C–E grades are more likely to result from a decline in pulmonary function, especially in individuals with current asthma or wheezing. Thus, data from these participants may have been undersampled. Finally, some important potential confounders such as allergic sensitization by environmental pollutants were not studied because these data were unavailable from the NHANES.

Overall, our study expands the understanding of the relationship between FI and respiratory health, particularly in adults, by providing data on the differential effects within specific populations. Future research should explore the underlying mechanisms of FI across different racial, sex, and age groups to gain a more comprehensive understanding of how FI impacts asthma. These insights will be crucial for developing more targeted public health interventions aimed at mitigating the adverse effects of FI on allergic diseases.

## Author Contributions

Weiliang Kong conceived and designed the study, and Weiliang Kong, Jingjing Hu, and Yilian Xie completed statistical analyses and analyzed the data. Weiliang Kong, Yilian Xie, Weina Huang, and Kunlong Xiong contributed to drafting and editing the paper. Weiliang Kong and Chao Cao had full access to all the data in the study and took responsibility for the integrity of the data and accuracy of the data analysis. All authors have given final approval of the manuscript.

## Ethics Statement

Detailed methods and protocols for the NHANES study were approved by the CDC/NCHS Research Ethics Review Board. They are publicly available through the CDC.gov website; this includes informed consent procedures for all participants. All methods in this study were performed by the relevant guidelines and regulations. This study was exempt from human subject ethical review as the data are freely available in the public domain.

## Consent

All the authors listed have approved the manuscript that is enclosed.

## Conflicts of Interest

The authors declare no conflicts of interest.

## Data Availability

The datasets analyzed for this study can be found in the CDC.gov website (https://www.cdc.gov/nchs/nhanes/index.htm).
